# Biochemical and kinetic characterisation of a novel xylooligosaccharide-upregulated GH43 β-d-xylosidase/α-l-arabinofuranosidase (BXA43) from the probiotic *Bifidobacterium animalis* subsp. *lactis* BB-12

**DOI:** 10.1186/2191-0855-3-56

**Published:** 2013-09-11

**Authors:** Alexander Holm Viborg, Kim Ib Sørensen, Ofir Gilad, Daniel Bisgaard Steen-Jensen, Adiphol Dilokpimol, Susanne Jacobsen, Birte Svensson

**Affiliations:** 1 Enzyme and Protein Chemistry, Department of Systems Biology, Technical University of Denmark, Søltofts Plads, Building 224, DK-2800 Kgs, Lyngby, Denmark; 2 Department for Strains, Chr. Hansen A/S, 2970 Hørsholm, Denmark; 3 Department for Identification, Chr. Hansen A/S, 2970 Hørsholm, Denmark

**Keywords:** Glycoside hydrolase family 43 (GH43), β-d-xylosidase, α-l-arabinofuranosidase, Xylooligosaccharides (XOS), Bifidobacteria, Probiotics

## Abstract

The *Bifidobacterium animalis* subsp. *lactis* BB-12 gene BIF_00092, assigned to encode a β-d-xylosidase (BXA43) of glycoside hydrolase family 43 (GH43), was cloned with a C-terminal His-tag and expressed in *Escherichia coli*. BXA43 was purified to homogeneity from the cell lysate and found to be a dual-specificity exo-hydrolase active on *para*-nitrophenyl-β-d-xylopyranoside (*p*NPX), *para*-nitrophenyl-α-L-arabinofuranoside (*p*NPA), β-(1 → 4)-xylopyranosyl oligomers (XOS) of degree of polymerisation (DP) 2–4, and birchwood xylan. A phylogenetic tree of the 92 characterised GH43 enzymes displayed five distinct groups (I − V) showing specificity differences. BXA43 belonged to group IV and had an activity ratio for *p*NPA:*p*NPX of 1:25. BXA43 was stable below 40°C and at pH 4.0–8.0 and showed maximum activity at pH 5.5 and 50°C. *K*_m_ and *k*_cat_ for *p*NPX were 15.6 ± 4.2 mM and 60.6 ± 10.8 s^-1^, respectively, and substrate inhibition became apparent above 18 mM *p*NPX. Similar kinetic parameters and catalytic efficiency values were reported for β-d-xylosidase (XynB3) from *Geobacillus stearothermophilus* T‒6 also belonging to group IV. The activity of BXA43 for xylooligosaccharides increased with the size and was 2.3 and 5.6 fold higher, respectively for xylobiose and xylotetraose compared to *p*NPX. BXA43 showed clearly metal inhibition for Zn^2+^ and Ag^+^, which is different to its close homologues. Multiple sequence alignment and homology modelling indicated that Arg^505^Tyr^506^ present in BXA43 are probably important for binding to xylotetraose at subsite +3 and occur only in GH43 from the *Bifidobacterium* genus.

## Introduction

Prebiotics are commonly non-digestible oligosaccharides which improve the composition of the gut microbiota thus eliciting beneficial health effects (Macfarlane et al. 2006; van den Broek et al. 2008). With a growing market for prebiotic containing foods there is increasing interest in understanding how prebiotics function at the molecular level. Two approved prebiotics are fructo-oligosaccharides (FOS) and galacto-oligosaccharides (GOS), while *β*-(1 → 4) linked xylo-oligosaccharides (XOS) with degree of polymerisation (DP) of 2–10 are considered emerging prebiotics (Roberfroid, 2007). Similarly to the approved prebiotics, FOS and GOS, XOS enhance growth of probiotic *Bifidobacterium* and *Lactobacillus* species, while suppressing *Bacteroides species* (Mäkeläinen et al. 2010) and pathogens, *e.g. Clostridium* species (Rycroft et al. 2001). Notably, XOS can lower the amount of secondary bile acids associated with potential tumour-promoting activity (Moure et al. 2006).

XOS are obtained by hydrolysis of xylans, which are linear *β*-(1 → 4) linked polysaccharides, typically decorated at the 2- and/or 3-position by mono- or di-substitution with *α*-l-arabinofuranosyl residues and a substitution pattern varying with the botanical origin (Ebringerova 2006). Probiotic bacteria can possess intracellular xylosidases degrading XOS to D-xylose, which in bifidobacteria is metabolised via the D-fructose-6-P shunt, also referred to as the bifid shunt (Ruas-Madiedo et al. 2005). We found a novel β-d-xylosidase BXA43 of glycoside hydrolase family 43 (GH43) (Cantarel et al. 2009) that was highly up-regulated in *Bifidobacterium animalis* subsp. *lactis* BB-12 (BB-12) cultures grown with XOS as the sole carbon source and probably plays an important role in the XOS catabolism (Gilad et al. 2010).

GH43 comprises a broad variety of enzyme specificities (Cantarel et al. 2009) encompassing β-d-xylosidase (EC 3.2.1.37), β-1,3-xylosidase (EC 3.2.1.-), α-l-arabinofuranosidase (EC 3.2.1.55), endo-arabinanase (EC 3.2.1.99), xylanase (EC 3.2.1.8), and galactan 1,3-β-galactosidase (EC 3.2.1.145) of which some are central in biomass utilisation in the biorefinery and bioenergy areas, while others from gut microbiomes of different animals play prominent roles in nutrition and dietary fibre utilisation (Wagschal et al. 2009b; Lagaert et al. 2011; Zhou et al. 2012). Eleven out of the 92 characterised GH43 enzymes are dual-function β-d-xylosidase-α-l-arabinofuranosidases (E.C. 3.2.1.37 / 3.2.1.55) accommodating at subsite −1 d-xylopyranose and l-arabinofuranose residues sharing the spatial orientation of sugar ring hydroxyl groups (Wagschal et al. 2009a). Other enzymes with this dual specificity occur in GH3, 51, and 54 (Jordan et al. 2007; Minic et al. 2004; Shallom et al. 2005; Xiong et al. 2007), however only GH43 uses an inverting mechanism (Armand et al. 1996; Pitson et al. 1996). GH43 and GH62 form clan GH-F (Cantarel et al. 2009) with characteristic 5-fold β-propeller structures available of GH43 β-d-xylosidase, α-l-arabinofuranosidase, arabinanase, and β-1,3-xylosidase (Brüx et al. 2006; Vandermarliere et al. 2009; de Sanctis et al. 2010; Cartmell et al. 2011).

The present study focuses on characterisation of the GH43 β-d-xylosidase/α-l-arabinofuranosidase BXA43 from *Bifidobacterium animalis* subsp. *lactis* BB-12 (BB-12) which was 10–30 fold upregulated in BB-12 grown on XOS as demonstrated by qPCR and DNA microarray analyses (Gilad et al. 2010). This dual-function GH43 was suggested to be important in the catabolism of XOS taken up by BB-12 to d-xylose. A comprehensive phylogenetic analysis of GH43 as well as a homology model provides a basis for the comparative analysis of the enzymatic properties of BXA43.

## Materials and methods

### Cloning

The BXA43 gene from BB-12 (locus tag BIF_00092, NCBI accession ADC85541), annotated to encode a β-d-xylosidase, was isolated and amplified from chromosomal DNA (provided by Chr. Hansen A/S, Hørshom, Denmark) using upstream primers designed to precede the native Shine-Dalgarno sequence and downstream primers complementary to the 3’-terminus and excluding the stop codon; *Bam*HI and *Xho*I sites are underlined.

Forward: BXA43-5′ CGCGGATCCAACCGGGCCGC CGTTTC

Reverse: BXA43-3′ CCG*C*TCGAGTTCACTCAATTC GCGGTAATC

The amplified product was cloned into the pET24(+) expression vector (Novagen) resulting in the plasmid pET24(+)-BXA43 encoding BXA43 with a C-terminal His-tag.

### Recombinant expression and purification

*Escherichia coli* BL21 (DE3) was transformed with pET24(+)-BXA43 and incubated overnight (agar plates, Luria-Bertani (LB), 25 μg/ml kanamycin, 37°C). Transformants were selected based on restriction site patterns and verified by DNA sequencing (Macrogen). A glycerol stock stab of pET24(+)-BXA43 in *E. coli* BL21(DE3) was propagated (20 ml LB medium, 50 μg/ml kanamycin, 37°C overnight) to inoculate LB (500 ml) to OD_600_ = 0.1, grown (37°C, 160 rpm) until OD_600_ = 0.6 and induced (3 h) by 100 μM isopropyl β-d-1-thiogalactopyranoside. The cells were harvested by centrifugation (3200 *g*, 20 min) and the pellet stored at −20°C. To purify BXA43 the pellet was resuspended and the cells lysed with 5 ml Bug Buster (Novagen) and 50 U/ml benzonase (Sigma-Aldrich) for 30 min at room temperature, cooled on ice, and centrifuged (as above) to remove debris. The supernatant was filtered (0.45 μm, Millipore), applied to a HisTrap™ HP (1 ml Ni-NTA column, GE Healthcare) equilibrated in 10 mM imidazole, 500 mM NaCl, 30 mM Tris/HCl, pH 8.0 and eluted by a linear gradient of 0–400 mM imidazole in the same buffer. Protein-containing fractions (monitored by A_280_) were pooled, dialysed against 50 mM Na-acetate pH 5.2 at 4°C overnight (10 kDa cut-off dialysis membrane; Millipore) and concentrated (10 kDa cut-off centrifugal filter; Millipore) to 2.4 mg/ml as determined by aid of amino acid analysis (Barkholt and Jensen 1989). The identity of BXA43 was verified by SDS-PAGE and by peptide mass fingerprinting (Ultraflex II TOF/TOF, Bruker Daltonics) (not shown).

### Effect of pH and temperature on stability and activity

The stability of 9.7 nM BXA43 was determined at pH 3–10 by incubation at 4°C for 24 h in Britton-Robinson’s universal buffers (Britton and Robinson, 1931) followed by measurement of the residual activity towards *p*NPX (see below). Thermostability of 9.7 nM BXA43 was determined by measuring activity towards *p*NPX after 10 min incubation at 20–65°C in 50 mM Na-acetate, pH 5.2. The temperature dependence of activity towards *p*NPX (see below) was determined in the same range. Half-lives of 9.8 nM BXA43 at 40–60°C were determined in 50 mM Na-acetate, pH 5.2 based on residual activity in aliquots (20 μl) removed at appropriate time intervals, followed by immediate addition of ice-cold 50 mM Na-acetate pH 5.2 (20 μl). Samples were kept on ice until assayed. Half-lives were calculated according to t_½_ = ln(2) / Ae^-Ea / RT^, where A is the pre-exponential factor, E_a_ the activation energy, R the gas constant, and T the temperature in Kelvin.

### Enzyme assays

The activity of BXA43 (9.7 nM) towards 1 mM *para*-nitrophenyl-β-d-xylopyranoside (*p*NPX; Sigma) or *para*-nitrophenyl-α-L-arabinofuranoside (*p*NPA; Sigma) was determined from released *para*-nitrophenol measured at 410 nm (ELISA reader; Powerwave XS; Holm & Halby). The substrate in 50 mM Na-acetate, pH 5.2 (40 μl) at 40°C was preincubated (5 min) and the reaction initiated by addition of BXA43 (10 μl) and terminated after 10 min by addition of 1 M Na_2_CO_3_ (100 μl). One unit was defined as the amount of enzyme (in mg) that releases 1 μmol *para*-nitrophenol/min. Enzymatic activity measurements were performed (in triplicate) according to this procedure, unless otherwise stated.

To determine kinetic parameters, initial rates of hydrolysis were obtained from five time points (0–16 min) and 14 *p*NPX concentrations (0.25–80 mM) using 7.8 nM BXA43 at the above conditions (in duplicates of four independent reactions). Kinetic parameters, *k*_cat_ (*V*_max_), *K*_m_, and *K*_i_ were calculated by curve fitting the data to the Michaelis-Menten equation describing substrate inhibition, *v* = *k*_*cat*_ x [E] x S / (*K*_m_ + S x (1 + S/*K*_i_)), using GraphPad Prism 5 (GraphPad Software).

Activity towards 1% (w/v) birchwood xylan, oat spelt xylan and rye arabinoxylan (all Megazyme) by 78 nM BXA43 was assessed at the above conditions (50 μl) after overnight incubation by thin layer chromatography (TLC; Silica gel 60 F254) of aliquots (1 μl) with arabinose and xylose as reference and developed in 8:2:1 ethyl-acetate:isopropanol:water for approx. 45 min and dried, followed by staining the carbohydrates with 2% (w/v) orcinol.

The specific activity on 2.25 mM xylobiose (*X*_2_), xylotriose (X_3_), and xylotetraose (X_4_) (all Megazyme) of 4.9 nM BXA43 was determined in 50 mM Na-acetate pH 5.2 (50 μl), at 40°C for 30 min using the dinitrosalicylic acid (DNS) reducing sugar assay monitored at 540 nm (Miller 1959) with xylose as standard.

The effect of 1 mM Ag^+^, Ca^2+^, Mg^2+^, Ni^2+^, or Zn^2+^ on activity towards *p*NPX of 9.8 nM BXA43 was determined essentially as above (in duplicate).

### Bioinformatics

Sequences were retrieved of the 92 annotated GH43 enzymes in the CAZy database (Cantarel et al. 2009) and their multiple sequence alignment (ClustalW2; Goujon et al. 2010) was visualised with ESPript (Gouet et al. 1999) and used to construct a phylogenetic tree (Dendroscope; Huson et al. 2007).

A homology model of BXA43 was built (HHPred; Söding et al. 2005) with XynB3 from *Geobacillus stearothermophilus* T-6 66% similarity, 50% identity; PDB: 2EXH, (Brüx et al. 2006) as template. X_4_ from the complex with *Bs*AXH-m2,3 from *Bacillus subtilis* subsp. *subtilis* str. 168 (PDB: 3C7G, Vandermarliere et al. 2009) was superimposed at subsites +1 through +4.

## Results

**Figure 1:**
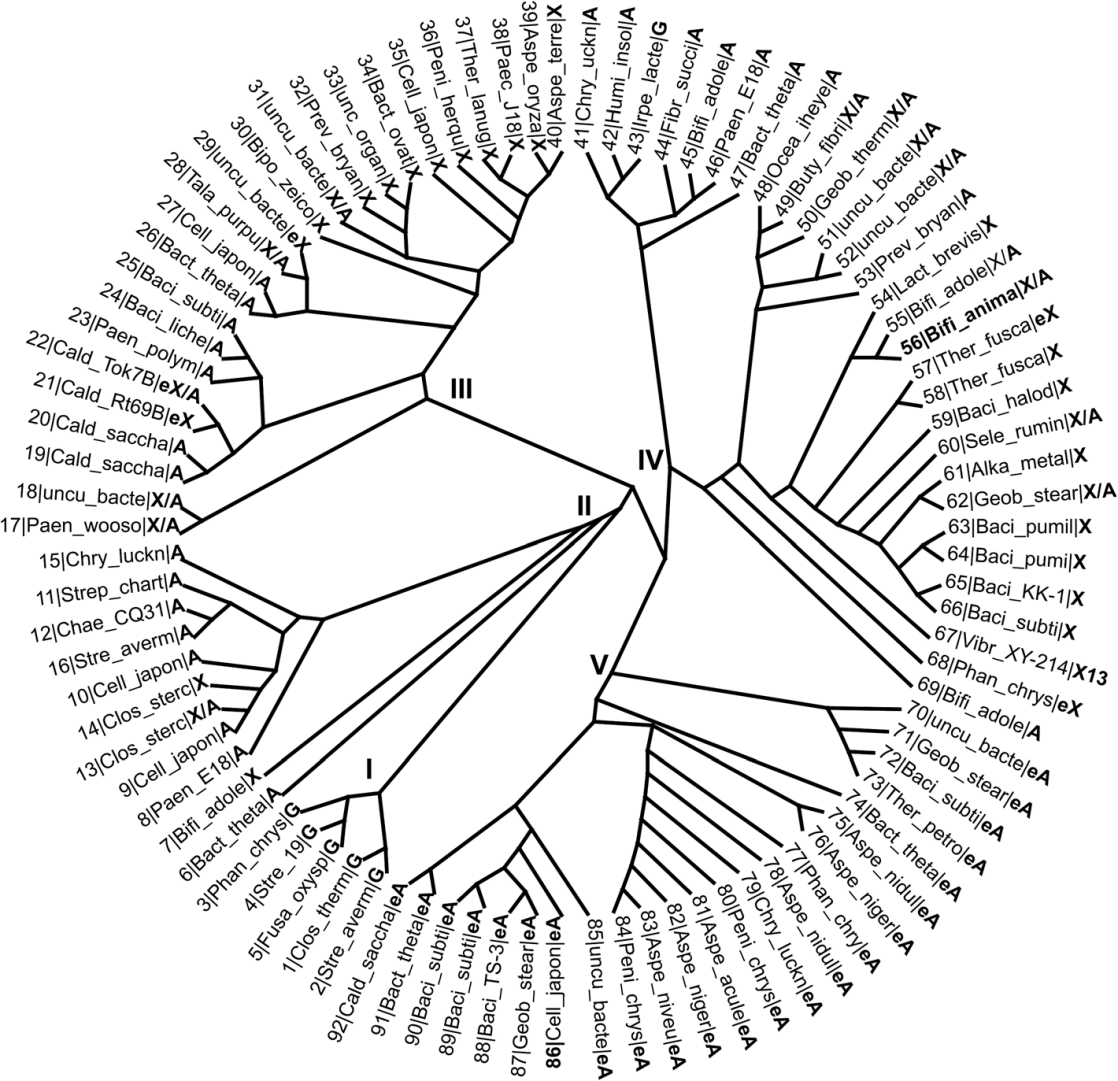
**Phylogenetic tree of GH43 enzymes annotated as characterised in the CAZy database (prepared by Dendroscope).** Five distinct groups (I-V) are observed which represent different specificity clusters. BXA43 (in **bold**) is found in group IV. Enzyme activities have been abbreviated as X: β-d-xylosidase (EC 3.2.1.37). X13: β-1,3-xylosidase (EC 3.2.1.-). A: α-l-arabinofuranosidase (EC 3.2.1.55). eA: endo-arabinanase (EC 3.2.1.99), eX: endo-xylanase (EC 3.2.1.8). G: galactan 1,3-β-galactosidase (EC 3.2.1.145). The entries are numbered and further details like the full organism names and UniProt identifiers can be found in Additional file 1: Table S1.

The *BXA43* gene from BB-12, annotated to encode a β-d-xylosidase of GH43 (Cantarel et al. 2009), has an ORF of 1626 nucleotides corresponding to 541 amino acid residues. GH43 (http://www.cazy.org; Cantarel et al. 2009) includes β-d-xylosidases (E.C. 3.2.1.37) and α-l-arabinofuranosidases (E.C. 3.2.1.55) and the phylogenetic tree of 92 GH43 enzymes, which are annotated as characterised in the CAZy database (Cantarel et al. 2009; Figure 1), shows five distinct groups (I − V) representing characteristic substrate specificities. Group I consists of galactan 1,3-β-galactosidases (EC 3.2.1.145), group II mainly of α-l-arabinofuranosidases (EC 3.2.1.55), while group III and IV are divided in two sub-groups, one of which contains predominately extracellular α-l-arabinofuranosidases (EC 3.2.1.55) and the other intracellular β-d-xylosidases (EC 3.2.1.37). Group V is solely composed of endo-arabinanases (EC 3.2.1.99).

Dual-specificity β-d-xylosidase/α-l-arabinofuranosidases are found in GH43 groups II, III, and IV and BXA43 belongs to group IV. The dual specificity β-d-xylosidase/α-l-arabinofuranosidase XylC from *B. adolescentis* ATCC 15703 is the closest well characterised relative 87% similarity, 79% identity; UniProt accession A1A0H6; (Lagaert et al. 2011), but BXA43 is also similar to another characterised group IV β-d-xylosidase/α-l-arabinofuranosidase SXA from *Selenomonas ruminantium* GA192 68% similarity, 52% identity; UniProt accession O52575; (Jordan et al. 2007). The crystal structure of a complex with xylobiose (PDB: 2EXH, 2EXJ, Brüx et al. 2006) is available for the group IV β-d-xylosidase XynB3 from *Geobacillus stearothermophilus* T-6 (66% similarity, 50% identity; UniProt accession Q09LX0). A multiple sequence alignment (Additional file 1: Figure S1) shows conservation in BXA43 of the general base and acid catalytic residues Asp^14^ and Glu^187^ as well as Asp^127^ proposed to modulate p*K*_a_ of the catalytic acid (Nurizzo et al. 2002), and various residues involved in substrate binding, including His^250^ at subsite −1 (Brüx et al. 2006). It also reveals that BXA43 Tyr^506^ and Arg^505^, which might be involved in the positioning of Tyr^506^, are not conserved in XynB3. These structural features may be important for the specificity of BXA43 in particular towards xylooligosaccharides.

**Figure 2:**
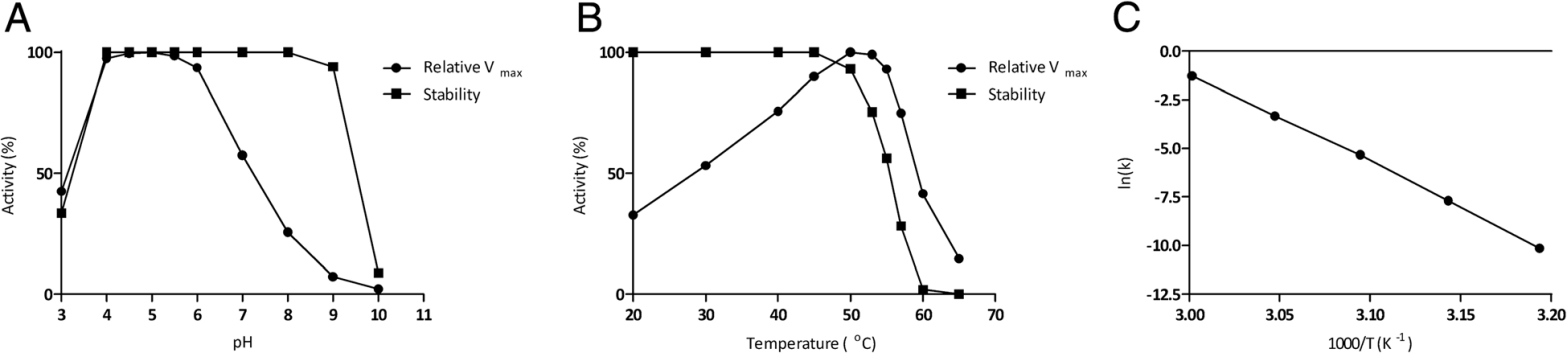
**Temperature and pH parameters of BXA43. (A)** Stability (*■*) and activity (relative *V*_max_) (*●*) pH profiles of BXA43 using *p*NPX as substrate. **(B)** Stability (***■***) and activity (relative *V*_max_) (***●***) pH profiles of BXA43 using *p*NPX as substrate. **(B)** Stability (■) and activity (relative *V*_max_) (●) of BXA43 as a function of temperature using *p*NPX as substrate. **(C)** Arrhenius plot of the effect of temperature on the t_½_ for thermal inactivation of BXA43 in the temperature range 40–60°C (see Methods for details).

Recombinant BXA43 was produced in *E. coli* and purified from the cell lysate in a final yield of 2.2 mg/L culture. It migrated in SDS-PAGE as a single band of approximately 62 kDa in excellent agreement with the theoretical mass of 61,774 Da. BXA43 was stable for 24 h at 4°C at pH 4–9 (Figure 2A). After 10 min at 45°C and 57°C, BXA43 retained 100% and 25% activity, respectively (Figure 2B). The t_½_ values of inactivation of 12 d at 40°C, 25.5 h at 45°C, 141 min at 50°C, 19.5 min at 55°C, and 2.5 min at 60°C indicated a linear correlation as described by the Arrhenius equation with activation energy, *E*_a_ = 384 kJ/mol (Figure 2C). These data suggest that BXA43 is very stable in the gut, its natural ecological niche.

**Figure 3:**
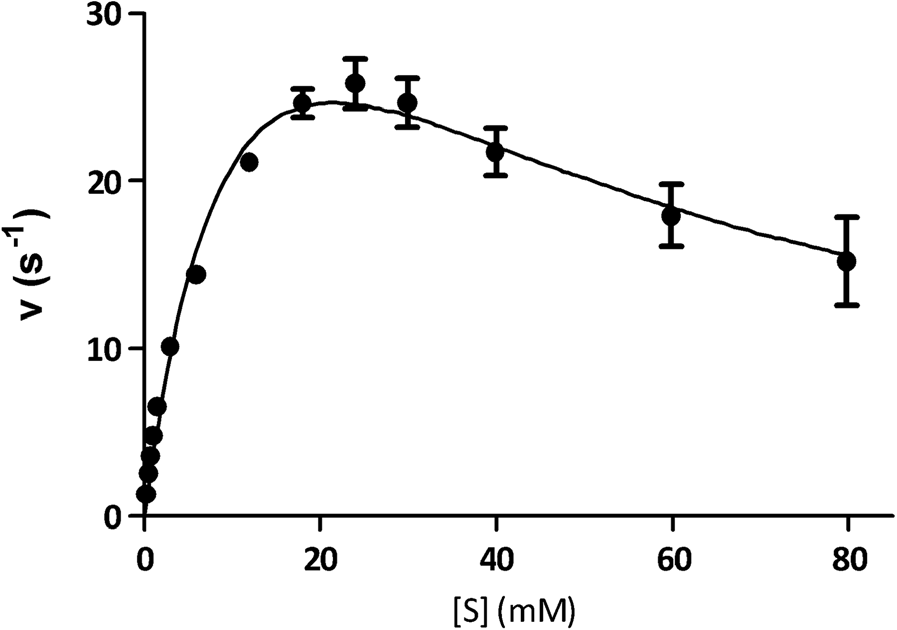
**Michaelis-Menten kinetics of BXA43 toward *p*NPX.** The fit is to uncompetitive substrate inhibition. All results are expressed as means ± standard error of the mean (SEM) n = 4 (see Methods for details).

BXA43 showed maximum activity towards *p*NPX at pH 4.0–5.5 and a temperature optimum of 50°C (Figure 2B). The kinetic parameters as derived from initial rates for hydrolysis of *p*NPX was *K*_m_ = 15.6 ± 4.2 mM and *k*_cat_ = 60.6 ± 10.8 s^-1^ assuming the presence of uncompetitive substrate inhibition with *K*_i_ = 29.6 ± 8.5 mM (Figure 3). BXA43 hydrolysed xylooligosaccharides and the specific activity for xylobiose (*X*_2_) was 2.3 fold higher than for *p*NPX and increased further for xylotriose (X_3_) and xylotetraose (X_4_) (Table 1). The activity towards *p*NPA was 4% of the value for *p*NPX, whereas *para*-nitrophenyl-β-d-galactopyranoside (*p*NPG) was not a substrate (Table 1). TLC indicated release by 20 μM BXA43 of xylose from birchwood xylan, but not from oat spelt xylan or rye arabinoxylan after 24 h incubation. The activity of BXA43 for *p*NPX decreased by 81% in the presence of 1 mM Zn^2+^, but only by 9% in 1 mM Ag^+^, while Ca^2+^, Mg^2+^ or Ni^2+^ did not affect the activity.

**Table 1:** **Specific activity of BXA43 towards *p*NP-monosaccharides and XOS**

**Substrate**	**U/mg**
*p*NPX	45.0 (100%)
*p*NPA	1.8 (4%)
*p*NPG	n.d.
Xylobiose (*X*_2_)	105 (233%)
Xylotriose (X_3_)	164 (364%)
Xylotetraose (X_4_)	253 (562%)

n.d. not detected.

## Discussion

BXA43 of *Bifidobacterium animalis* subsp. *lactis* BB-12 from GH43 was produced recombinantly in *E. coli* BL21 (DE3) and showed *K*_m_ of 15.6 ± 4.6 mM, *k*_cat_ of 60.6 ± 10.8 s^-1^ and the catalytic efficiency *k*_cat_/*K*_m_ of 3.9 s^-1^ mM^-1^ similar to *k*_cat_ of 57 s^-1^ and *k*_cat_/*K*_m_ of 3.3 s^-1^ mM^-1^ reported for β-xylosidase (XynB3) from *Geobacillus stearothermophilus* T‒6 (Brüx et al. 2006). By contrast, β-xylosidase/α-l-arabinofuranosidase from *S. ruminantium* GA192 has much lower *K*_m_ = 0.38 mM (Brunzelle et al. 2008), and a β-xylosidase from *Bacillus pumilus* IPS has *K*_m_ = 3.9 mM (Xu et al. 1991). Remarkably, *V*_max_ of BXA43 of 166 U mg^-1^ was 11-fold higher than *V*_max_ of 15 U mg^-1^ obtained for the GH43 enzyme from the thermophile *Clostridium stercorarium* F-9 (Suryani et al. 2004). BXA43 showed decreased rate of hydrolysis above 18 mM *p*NPX indicating apparent substrate inhibition (*K*_i_ = 29.6 ± 8.5 mM), which however, was not a result of product condensation as verified by TLC (data not shown) consistent with no reports of condensation activity of GH43 enzymes. By contrast, the retaining *Thermoanaerobacter ethanolicus* β-xylosidase/α-l-arabinofuranosidase from GH3 was subject to substrate inhibition at low substrate concentration of 0.5 mM *p*NPX (Mai et al. 2000).

As BXA43 has 5°C lower temperature optimum than the group IV enzyme deXA isolated from a compost starter mixture (Wagschal et al. 2009a) the thermal half-lives determined of BXA43 (141 min at 50°C; 19.5 min at 55°C; 2.5 min at 60°C) and deXA (630 min at 49°C; 234 min at 51°C; 47 min at 53°C) are comparable. BXA43 was inhibited by 1 mM Zn^2+^ and less sensitive to Ag^+^, while a GH43 β-d-xylosidase from *Talaromyces thermophilus* was inhibited by both Zn^2+^, Cu^2+^ and Hg^2+^ (Guerfali et al. 2008). Remarkably, 1 mM Zn^2+^ had no effect on activity of the β-d-xylosidase/α-l-arabinofuranosidase from *G. stearothermophilus* T-6, which has 66% sequence identity and similar specific activity to BXA43, whereas 1 mM Ag^+^ inactivated this enzyme (Shallom et al. 2005). Thus, distinctly different metal ion sensitivity is observed among GH43 enzymes.

**Figure 4:**
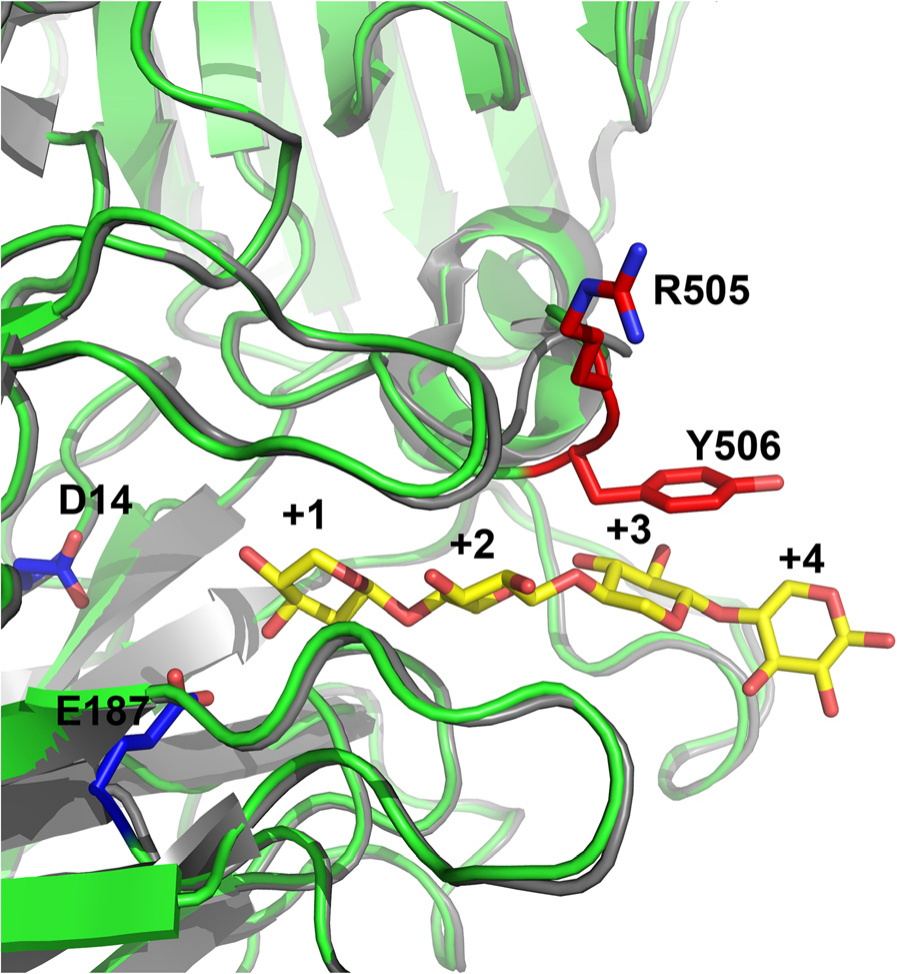
**Model of BXA43 (green) superimposed with the structure of the close homolog XynB3 (grey, 2EXH), showing the two catalytic residues D**^**14 **^**and E**^**187 **^**(in blue; BXA43 numbering).** Xylotetraose (yellow) was manually fitted from the structure of the complex with *Bs*AXH-m2,3 on *Bacillus subtilis* (grey, 3C7G) (Vandermarliere *et al*., 2009) occupying the +1, +2, +3, and +4 subsites and illustrating a predicted +3 subsite involvement of Y^506^ (red). Arg^505^ in BXA43 may play a role in the positioning of Tyr^506^.

BXA43 hydrolysed *X*_2_, X_3_, and X_4_ in line with a suggested model for XOS utilisation in BB-12 that includes uptake *via* an ATP binding cassette (ABC) oligosaccharide transport system whose components were identified in the secreted (secretome) and the membrane proteomes of BB-12 grown on XOS; BB-12 is incapable of growing on xylose as the sole carbon source (Gilad et al. 2011; 2012). While an increasing specific activity for *X*_2_, X_3_, and X_4_ (Table 1) is seen for BXA43, this was not reported for GbtXyl43A from *G. thermoleovorans* IT-08 (Wagschal et al. 2009b) and deAX from an uncultured bacterium (Wagschal et al. 2009a) which both belong to group IV and have the highest specific activity on *X*_2_ or X_3_, respectively. This can possibly be explained by the uniqueness of the BXA43 Tyr^506^ residue, found only within the *Bifidobacterium* and *Lactobacillus* GH43 enzymes. This residue may stack onto substrate at subsite +3 in BXA43, as supported by homology modelling (Figure 4). Interestingly, the group IV XylC from *B. adolescentis* ATCC 15703 that is hypothesised to play an important role in the efficient conversion of *X*_2_ to xylose (Lagaert et al. 2011), possesses the corresponding tyrosine residue, has slightly higher specific activity on *X*_2_ (23.7 s^-1^ mM^-1^) than X_4_ (17.0 s^-1^ mM^-1^), and the highest specific activity observed towards xylohexaose (X_6_, 24.6 s^-1^ mM^-1^). Notably, *B. adolescentis* ATCC 15703 also harbours a GH120 β-d-xylosidase (XylB), not found in BB-12, which shows a drastic increase in specific activity from *X*_2_ (0.7 s^-1^ mM^-1^) to X_4_ (17.6 s^-1^ mM^-1^,Lagaert et al. 2011).

A phylogenetic tree (Figure 1) generated for characterised GH43 enzymes, as annotated by the CAZy database (Cantarel et al. 2009) of which approximately 35% have been studied and 13 have a known *p*NPX:*p*NPA activity ratio (Table 2), has five clusters (groups I − V) (Figure 1) representing distinct substrate specificities, as opposed to a previously reported phylogenetic tree (Qian et al. 2003) that displayed four groups. The two phylogenetic trees, however, are based on different dataset and are analysed with different purposes, therefore their group numbering is not directly comparable. The newly updated tree provides more specific grouping, most likely due to the better current annotation of the GH43 genes, as biochemical data have been reported for all proposed five phylogenetic groups after the previous tree was published.

**Table 2:** ***p*NPX: *p*NPA activity ratio of biochemically characterised dual-function β-d-xylosidases/α-l-arabinofuranosidases from GH43**

**Enzyme name ** **Organism ** **(UniProt identifier)**	***p*NPX: *p *NPA** **ratio**	**Phylogenetic** **group**^a^	**Reference**
*C. stercorarium* (P48790)	2.5	II	Sakka et al. 1993
RunXyn1 Uncultured rumen bacterium (E3TBJ3)	2.6	III	Zhou et al. 2012
r_09-02 uncultured bacterium r_09 (I6YTF5)	1.1	III	Ferrer et al. 2012
XylC *Paenibacillus woosongensis* (E7DXB6)	1.9	III	Kim and Yoon 2010
ABF3 *P. purpurogenum* (C3W4Y2)	5.2	III	Ravanal et al. 2010
BXA43 *B. animalis* subsp. *lactis* BB-12 (D3R467)	25.0	IV	This study
XynB3 *G. stearothermophilus* T-6 (Q09LX0)	22.2	IV	Shallom et al. 2005
SXA *S. ruminantium* GA192 (O52575)	12.7	IV	, 2001
XylC *B. adolescentis* ATCC 15703 (A1A0H6)	9.1	IV	Lagaert et al. 2011
XylB *B. fibrisolvens* GS 113 (P45982)	0.6	IV	Utt et al. 1991
deAFc Uncultured bacterium (A0S5D8)	0.6	IV	Wagschal et al. 2007
deAX Uncultured bacterium (B8QP77)	0.5	IV	Wagschal et al. 2009a
GbtXyl43A *G. thermoleovorans* IT-08 (Q2I2N4)	0.1	IV	Wagschal et al. 2009b

^a^This study.

BXA43 is classified as a dual-function β-xylosidase/α-l-arabinofuranosidase of group IV (Figure 1). Dual-function β-xylosidases/α-l-arabinofuranosidases occur in groups II, III and IV and their *p*NPX:*p*NPA activity ratios vary in the range 0.1–25 (Table 2), with BXA43 having highest and almost the same ratio as XynB3 from *G. stearothermophilus* T-6 (Shallom et al. 2005). The relatively low activity of BXA43 for *p*NPA suggests, as previously hypothesised (Gilad et al. 2010), a main role of BXA43 in intracellular hydrolysis of XOS. BLASTP searches revealed that only enzymes of the *Bifidobacterium* genus contain both Arg^505^ and Tyr^506^ proposed to be implicated in high activity on xylooligosaccharides. One gene, however, from *Lactobacillus brevis* ATCC 367 (ABJ65333.1, Makarova et al. 2006) from group IV, is annotated as β-xylosidase and encodes a tyrosine in the same position as Tyr^506^, but the corresponding protein has not been described.

BXA43 was able to release xylose from birchwood xylan, but not from oat spelt xylan or rye arabinoxylan. While arabinose substituents increase the bulkiness of xylans and can interfere with their accommodation in the active site of enzymes from GH43, it appears that α-(1 → 2)-4-*O*-methyl-d-glucuronic acid substituents on birchwood xylan are oriented in a way that allows BXA43 to hydrolyse the release of a xylosyl residue preceding to a substituted backbone xylosyl residue (Tenkanen et al. 1996). Arabinose was not detected after incubation of oat and rye arabinoxylans with BXA43, thus the active site seemed unable to accommodate arabinose substituents of these polysaccharides in a productive manner at subsite −1 despite its clear activity on *p*NPA. A similar observation is reported for XylC from *B. adolescentis* ATCC 15703 (Lagaert et al. 2011). However without structural data on these enzymes and their interaction with *e.g.* xylotetraose at the active site it is not readily explained why they do not release arabinose from oat and rye arabinoxylans.

## Competing interests

The authors declare that they have no competing interests.

## Supplementary Material

Additional file 1: Figure S1 Multiple sequence alignment using ESPript (Gouet et al. 1999) of GH43 phylogenetic group IV, including BXA43 (ADC85541.1) and XynB3 (AAT98625.1), reveals that BXA43 R^505^ and Y^506^ (red stars), which are located in the spatial proximity of the active site, are not conserved in XynB3. BAF39209.1 from *Bifidobacterium adolescentis* ATCC 15703 illustrates the conserved R^505^ and Y^506^ in Bifidobacteria. Catalytic residues (green stars) and the proposed histidine (Brüx et al. 2006) involved in subsite –1 interaction (blue star). **Table S1.** Organisms associated with GenBank accessions in Figure 1. Click here for file
