# Supplementary material for: Biochemical and kinetic characterisation of a novel xylooligosaccharide-upregulated GH43 β-d-xylosidase/α-l-arabinofuranosidase (BXA43) from the probiotic Bifidobacterium animalis subsp. lactis BB-12
Source: AMB Express. 2013 Sep 11;3:56. doi: 10.1186/2191-0855-3-56 (PMC3847938; doi:10.1186/2191-0855-3-56)
Supplement: Additional file 1: Figure S1 — Multiple sequence alignment using ESPript (Gouet et al. 1999) of GH43 phylogenetic group IV, including BXA43 (ADC85541.1) and XynB3 (AAT98625.1), reveals that BXA43 R505 and Y506 (red stars), which are located in the spatial proximity of the active site, are not conserved in XynB3. BAF39209.1 from Bifidobacterium adolescentis ATCC 15703 illustrates the conserved R505 and Y506 in Bifidobacteria. Catalytic residues (green stars) and the proposed histidine (Brüx et al. 2006) involved in subsite –1 interaction (blue star). Table S1. Organisms associated with GenBank accessions in Figure 1. [file 2191-0855-3-56-S1.docx]

**Supplementary materials**

**Biochemical and kinetic characterisation of a novel xylooligosaccharide-upregulated GH43 β-d-xylosidase/α-l-arabinofuranosidase (BXA43) from the probiotic *Bifidobacterium animalis* subsp. *lactis* BB-12**

**Alexander Holm Viborg^1^, Kim Ib Sørensen^2^, Ofir Gilad^1,3^, Daniel Bisgaard Steen-Jensen^1^, Adiphol Dilokpimol^1^, Susanne Jacobsen^1^, and Birte Svensson^1*^**

Enzyme and Protein Chemistry, Department of Systems Biology, Technical University of Denmark, 2800 Kgs. Lyngby, Denmark^1^; Department for Strains, Chr. Hansen A/S, 2970 Hørsholm, Denmark^2^; Department for Identification, Chr. Hansen A/S, 2970 Hørsholm, Denmark^3^

**^*^Corresponding author:**

Birte Svensson

Enzyme and Protein Chemistry, Department of Systems Biology

Technical University of Denmark

Søltofts Plads, Building 224

DK-2800 Kgs. Lyngby, Denmark

Tel.: (+45) 4525 2740

Fax: (+45) 4588 6307

E-mail: bis@bio.dtu.dk


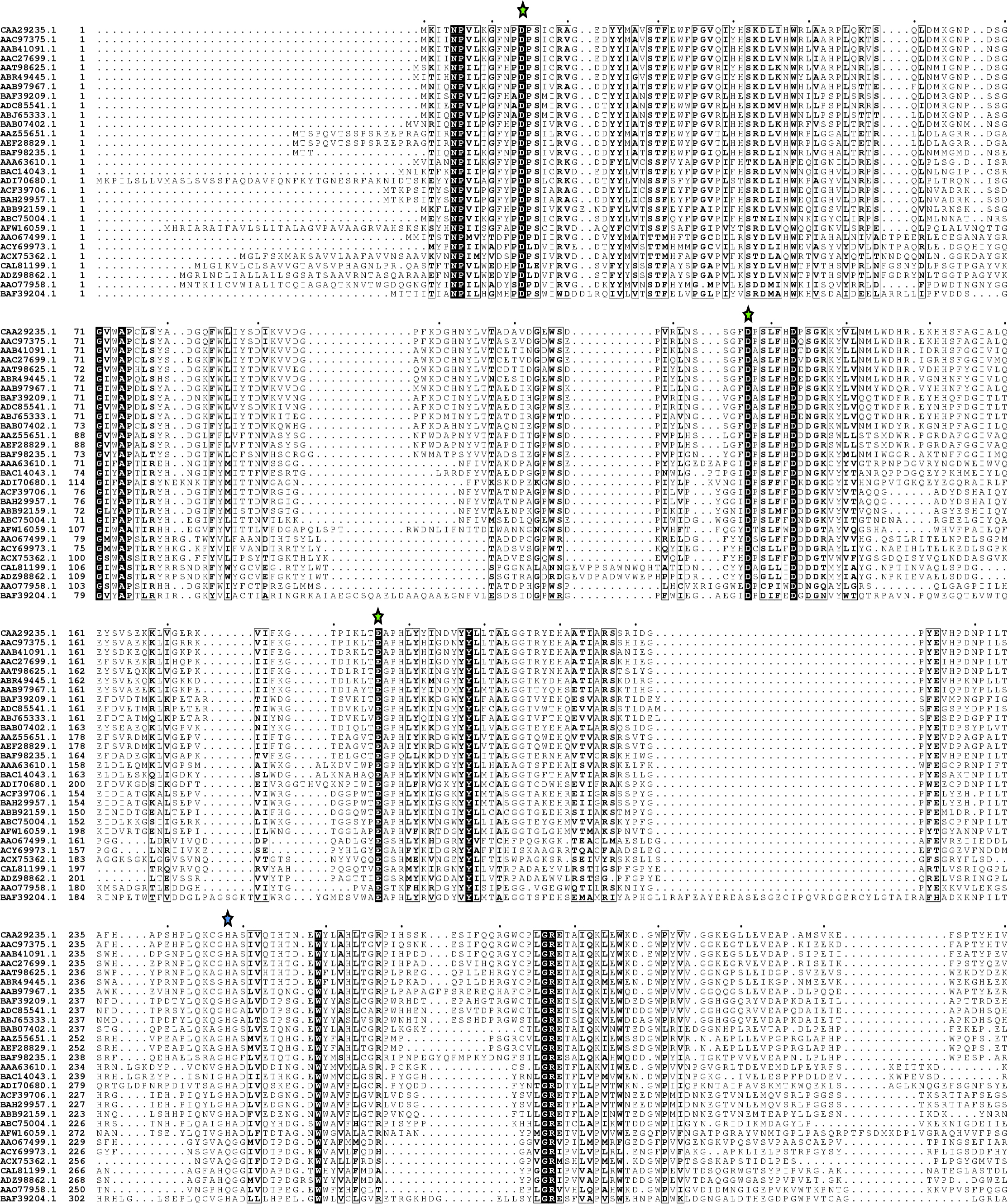


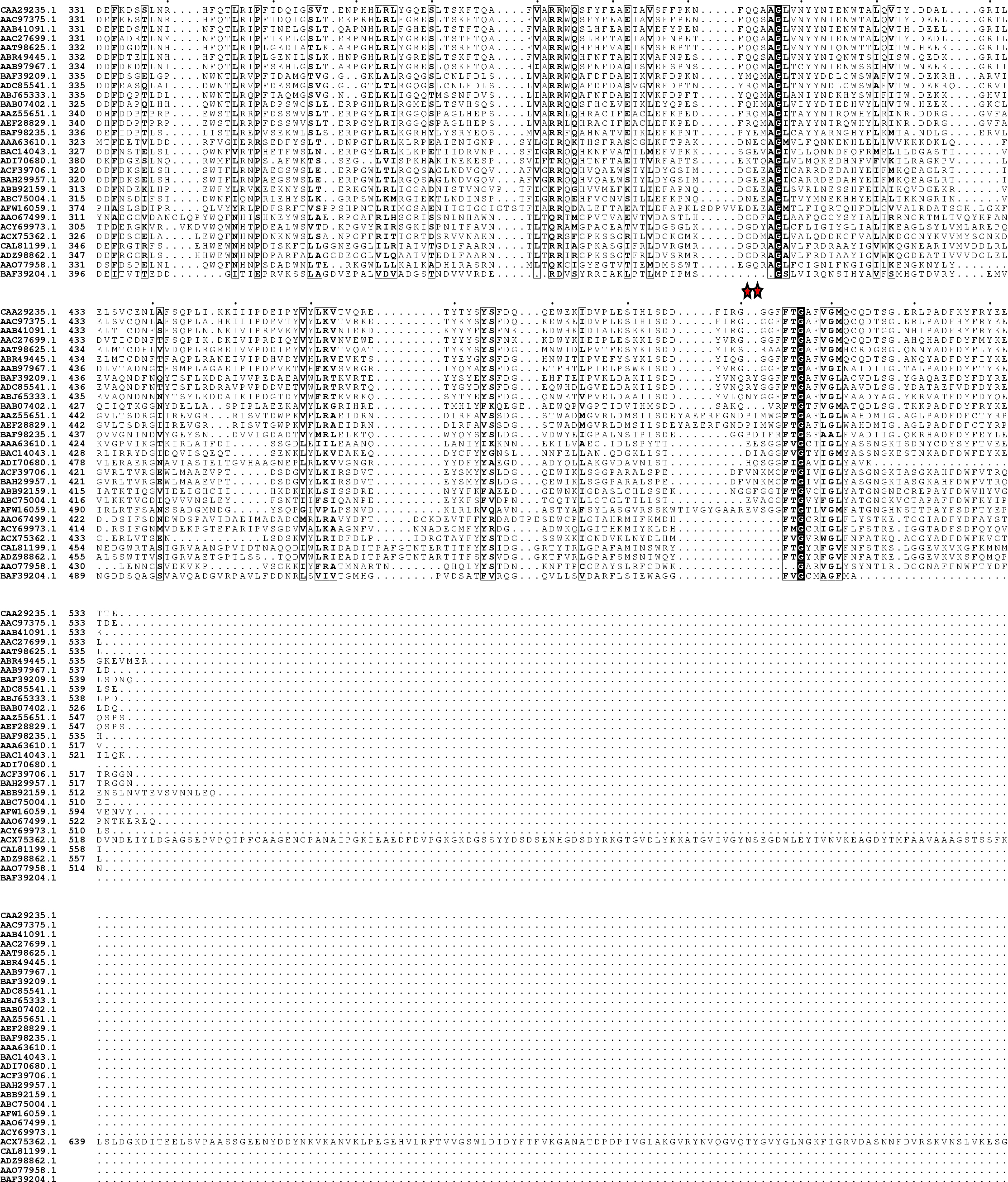


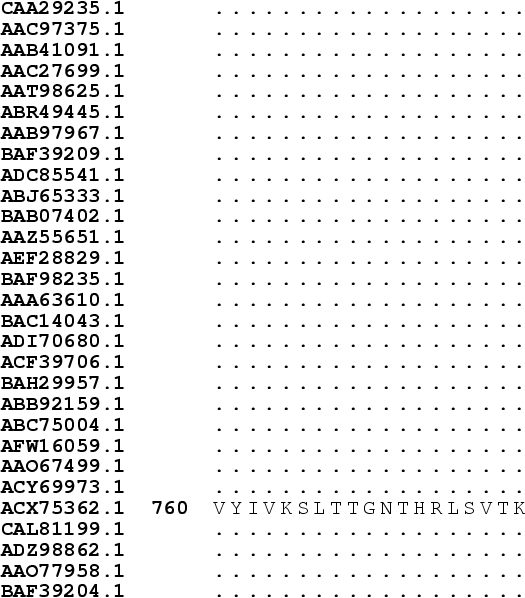


**Supplementary Fig S1** Multiple sequence alignment using ESPript (Gouet et al. 1999) of GH43 phylogenetic group IV, including BXA43 (ADC85541.1) and XynB3 (AAT98625.1), reveals that BXA43 R^505^ and Y^506^ (red stars), which are located in the spatial proximity of the active site, are not conserved in XynB3. BAF39209.1 from *Bifidobacterium adolescentis* ATCC 15703 illustrates the conserved R^505^ and Y^506^ in Bifidobacteria. Catalytic residues (green stars) and the proposed histidine (Brüx et al. 2006) involved in subsite –1 interaction (blue star).

**Supplementary Table S1** Organisms associated with GenBank accessions in Fig 1

| **Group** | **Tree Id** | **GenBank**  **Accession** | **UniProt**  **Identifier** | **Organism** |
| --- | --- | --- | --- | --- |
| 1 | 1 | ABN51896.1 | A3DD67 | *Clostridium thermocellum* ATCC 27405 |
| 1 | 2 | BAC69820.1 | Q82LA2 | *Streptomyces avermitilis* MA-4680 |
| 1 | 3 | BAD98241.1 | Q50KB2 | *Phanerochaete chrysosporium* |
| 1 | 4 | AFH55135.1 | I0B0T0 | *Streptomyces* sp. 19 |
| 1 | 5 | BAG80558.1 | B6F260 | *Fusarium oxysporum* |
|  |  |  |  |  |
| 2 | 6 | AAO78760.1 | Q8A1K6 | *Bacteroides thetaiotaomicron* VPI-5482 |
| 2 | 7 | BAF40308.1 | A1A3M5 | *Bifidobacterium adolescentis* ATCC 15703 |
| 2 | 8 | AFC38437.1 | J9PXM5 | *Paenibacillus* sp. E18 |
| 2 | 9 | ACE84379.1 | B3PKP9 | *Cellvibrio japonicus* Ueda107 |
| 2 | 10 | ACE82749.1 | B3PD54 | *Cellvibrio japonicus* Ueda107 |
| 2 | 11 | BAA90772.1 | P82594 | *Streptomyces chartreusis* |
| 2 | 12 | AFU88757.1 | K7QK60 | *Chaetomium* sp. CQ31 |
| 2 | 13 | BAA02527.1 | P48790 | *Clostridium stercorarium* |
| 2 | 14 | BAC87941.1 | Q76EC8 | *Clostridium stercorarium* |
| 2 | 15 | ADZ98860.1 | F2X2F6 | *Chrysosporium lucknowense* |
| 2 | 16 | BAC68753.1 | Q82P90 | *Streptomyces avermitilis* MA-4680 |
|  |  |  |  |  |
| 3 | 17 | ADV16404.1 | E7DXB6 | *Paenibacillus woosongensis* |
| 3 | 18 | AFN57709.1 | I6YTF5 | uncultured bacterium r_09 |
| 3 | 19 | AAB87371.1 | O30426 | *Caldicellulosiruptor saccharolyticus* |
| 3 | 20 | ABP67988.1 | A4XM53 | *Caldicellulosiruptor saccharolyticus* DSM 8903 |
| 3 | 21 | AAB95326.1 | O52374 | *Caldicellulosiruptor* sp. Rt69B.1 |
| 3 | 22 | AAD30363.1 | Q9X3P5 | *Caldicellulosiruptor* sp. Tok7B.1 |
| 3 | 23 | CAA40378.1 | P45796 | *Paenibacillus polymyxa* |
| 3 | 24 | BAL45491.1 | H1ACZ8 | *Bacillus licheniformis* |
| 3 | 25 | CAB13699.1 | Q45071 | *Bacillus subtilis* subsp. *subtilis* str. 168 |
| 3 | 26 | AAO75476.1 | Q8AAU5 | *Bacteroides thetaiotaomicron* VPI-5482 |
| 3 | 27 | ACE83886.1 | B3PD60 | *Cellvibrio japonicus* Ueda107 |
| 3 | 28 | ACP50519.2 | C3W4Y2 | *Talaromyces purpurogenus* |
| 3 | 29 | ACM91046.1 | C0K016 | uncultured bacterium URE4 |
| 3 | 30 | AAC67554.1 | O93912 | *Bipolaris zeicola* |
| 3 | 31 | ADO20354.1 | E3TBJ3 | uncultured rumen bacterium |
| 3 | 32 | CAA89208.1 | P48791 | *Prevotella bryantii* |
| 3 | 33 | AFP23142.1 | I7EUE2 | uncultured organism |
| 3 | 34 | AAB08024.1 | P49943 | *Bacteroides ovatus* |
| 3 | 35 | ACE82692.1 | B3PDB2 | *Cellvibrio japonicus* Ueda107 |
| 3 | 36 | BAC75546.1 | Q870E8 | *Penicillium herquei* |
| 3 | 37 | ADW66247.1 | G9B187 | *Thermomyces lanuginosus* |
| 3 | 38 | ADM33794.1 | F1APW0 | *Paecilomyces* sp. J18 |
| 3 | 39 | BAE55732.1 | Q2URT3 | *Aspergillus oryzae* RIB40 |
| 3 | 40 | AFD63137.1 | H9BYY0 | *Aspergillus terreus* |
|  |  |  |  |  |
| 4 | 41 | ADZ98862.1 | F2X2F8 | *Chrysosporium lucknowense* |
| 4 | 42 | CAL81199.1 |  | *Humicola insolens* |
| 4 | 43 | BAH29957.1 | B9ZZS1 | *Irpex lacteus* |
| 4 | 44 | ACX75362.1 | C9RS26 | *Fibrobacter succinogenes* subsp. *succinogenes* S85 |
| 4 | 45 | AAO67499.1 | Q5JB56 | *Bifidobacterium adolescentis* |
| 4 | 46 | ACY69973.1 | D6BQP5 | *Paenibacillus* sp. E18 |
| 4 | 47 | AAO77958.1 | Q8A3V2 | *Bacteroides thetaiotaomicron* VPI-5482 |
| 4 | 48 | BAC14043.1 | Q8EPL4 | *Oceanobacillus iheyensis* HTE831 |
| 4 | 49 | AAA63610.1 | P45982 | *Butyrivibrio fibrisolvens* |
| 4 | 50 | ABC75004.1 | Q2I2N4 | *Geobacillus thermoleovorans* |
| 4 | 51 | ABB92159.1 | A0S5D8 | uncultured bacterium |
| 4 | 52 | ACF39706.1 | B8QP77 | uncultured bacterium |
| 4 | 53 | ADI70680.1 | D7SFH6 | *Prevotella bryantii* B14 |
| 4 | 54 | ABJ65333.1 | Q03N89 | *Lactobacillus brevis* ATCC 367 |
| 4 | 55 | BAF39209.1 | A1A0H6 | *Bifidobacterium adolescentis* ATCC 15703 |
| 4 | 56 | ADC85541.1 | D3R467 | *Bifidobacterium animalis* subsp. *lactis* BB-12 |
| 4 | 57 | AEF28829.1 | F6LAX2 | *Thermobifida fusca* TM51 |
| 4 | 58 | AAZ55651.1 | Q47PG8 | *Thermobifida fusca* YX |
| 4 | 59 | BAB07402.1 | Q9K6P5 | *Bacillus halodurans* C-125 |
| 4 | 60 | AAB97967.1 | O52575 | *Selenomonas ruminantium* GA192 |
| 4 | 61 | ABR49445.1 | A6TTC7 | *Alkaliphilus metalliredigens* QYMF |
| 4 | 62 | AAT98625.1 | Q09LX0 | *Geobacillus stearothermophilus* |
| 4 | 63 | CAA29235.1 | P07129 | *Bacillus pumilus* |
| 4 | 64 | AAC97375.1 | Q9Z477 | *Bacillus pumilus* |
| 4 | 65 | AAC27699.1 | O52729 | *Bacillus* sp. KK-1 |
| 4 | 66 | AAB41091.1 | P94489 | *Bacillus subtilis* |
| 4 | 67 | BAF98235.1 | A9ZND1 | *Vibrio* sp. XY-214 |
| 4 | 68 | AFW16059.1 | K7T9D9 | *Phanerochaete chrysosporium* |
| 4 | 69 | BAF39204.1 | A1A0H1 | *Bifidobacterium adolescentis* ATCC 15703 |
|  |  |  |  |  |
| 5 | 70 | ADB43998.1 | D2XML7 | uncultured bacterium |
| 5 | 71 | ACE73680.1 | B3EYN2 | *Geobacillus stearothermophilus* |
| 5 | 72 | CAB15969.2 | P42293 | *Bacillus subtilis* subsp. *subtilis* str. 168 |
| 5 | 73 | ABQ46657.1 | A5IKD4 | *Thermotoga petrophila* RKU-1 |
| 5 | 74 | AAO75467.1 | Q8AAV4 | *Bacteroides thetaiotaomicron* VPI-5482 |
| 5 | 75 | EAA58736.1 | Q5AZC8 | *Aspergillus nidulans* FGSC A4 |
| 5 | 76 | CAK49041.1 | A2QT85 | *Aspergillus niger* |
| 5 | 77 | AFN42888.1 | I6XPK9 | *Phanerochaete chrysosporium* |
| 5 | 78 | EAA58810.1 | Q5AUM3 | *Aspergillus nidulans* FGSC A4 |
| 5 | 79 | ADZ98858.1 | F2X2F4 | *Chrysosporium lucknowense* |
| 5 | 80 | BAD15018.1 | Q75WE6 | *Penicillium chrysogenum* |
| 5 | 81 | AAG27441.1 | Q9HFS9 | *Aspergillus aculeatus* |
| 5 | 82 | AAA32682.1 | P42256 | *Aspergillus niger* |
| 5 | 83 | AEV23010.1 | H6TQN0 | *Aspergillus niveus* |
| 5 | 84 | BAD89094.2 | Q5H7M8 | *Penicillium chrysogenum* |
| 5 | 85 | ADB43999.1 | D2XML8 | uncultured bacterium |
| 5 | 86 | ACE84667.1 | B3PKP8 | *Cellvibrio japonicus* Ueda107 |
| 5 | 87 | ACE73676.1 | B3EYM8 | *Geobacillus stearothermophilus* |
| 5 | 88 | BAB64339.1 | Q93HT9 | *Bacillus* sp. TS-3 |
| 5 | 89 | BAA20372.1 | O07078 | *Bacillus subtilis* |
| 5 | 90 | CAA99586.1 | P94522 | *Bacillus subtilis* |
| 5 | 91 | AAO75474.1 | Q8AAU7 | *Bacteroides thetaiotaomicron* VPI-5482 |
| 5 | 92 | ABP67152.1 | A4XJR7 | *Caldicellulosiruptor saccharolyticus* DSM 8903 |
